# Supplementary material for: Geographical Discrimination in Curcuminoids Content of Turmeric Assessed by Rapid UPLC-DAD Validated Analytical Method
Source: Molecules. 2019 May 10;24(9):1805. doi: 10.3390/molecules24091805 (PMC6540245; doi:10.3390/molecules24091805)
Supplement: Supplementary file 1 [file molecules-24-01805-s001.pdf]

molecules

MDPI

Article

Geographical Discrimination in Curcuminoids Content of Turmeric Assessed by Fast UPLC-DAD Validated Analytical Method

Amrit Poudel [1,2]*, Jitendra Pandey [1] and Hyeong-Kyu Lee [1]

[1] Natural Medicine Research Center, Korea Research Institute of Bioscience and Biotechnology, Cheongju-si, 363-883, South Korea; amritpoudel@gmail.com (A.P.); jitupandey01301@gmail.com (J.P.); hykylee@kribb.re.kr (H.K.L)

[2] Department of Biodiversity and Bioresources, Satvik Nepal, Dadakonak-27, Pokhara, Nepal

* Correspondence: amritpoudel@gmail.com

Received: date; Accepted: date; Published: date

Supplementary Materials

Table S1. Robustness of instrument, volume of injection, column temperature and flow rate

Instrument	Standards	RT	N	K'	α	TF	Rs
Waters Aquity	Cur	0.701	6476.13	1.93		1.25	
	DMCur	0.800	8260.23	2.27	1.18	1.15	2.81
	BDMcur	0.902	8883.97	2.90	1.27	1.13	3.10
Thermo Scientific	Cur	0.663	3789.23	1.98		1.05	
	DMCur	0.787	5248.54	2.45	1.24	1.07	2.85
	BDMcur	0.930	6289.65	3.14	1.28	1.04	3.26
Agilent	Cur	0.671	3547.35	1.99		0.98	
	DMCur	0.788	5012.89	2.59	1.30	1.11	2.21
	BDMcur	0.922	6047.82	3.12	1.20	1.02	3.08
Volume of Injection							
5 μL	Cur	0.701	6476.13	1.93		1.25	
	DMCur	0.800	8260.23	2.27	1.18	1.15	2.81
	BDMcur	0.902	8883.97	2.90	1.27	1.13	3.10
3 μL	Cur	0.702	6403.08	2.04		1.30	
	DMCur	0.799	8176.16	2.46	1.21	1.18	2.79
	BDMcur	0.905	8895.65	2.90	1.18	1.13	3.06
7 μL	Cur	0.700	6087.35	2.06		1.17	
	DMCur	0.797	7854.38	2.48	1.20	1.10	2.22
	BDMcur	0.909	8715.85	2.96	1.20	1.09	2.59
Flow rate							
1 mL/min	Cur	0.701	6476.13	1.93		1.25	
	DMCur	0.800	8260.23	2.27	1.18	1.15	2.81
	BDMcur	0.902	8883.97	2.90	1.27	1.13	3.10

0.9 mL/min	Cur	0.768	6484.49	1.84		1.33	
	DMCur	0.872	8832.89	2.23	1.21	1.18	2.69
	BDMcur	0.992	9367.55	2.67	1.20	1.17	2.99
1.1 mL/min	Cur	0.642	5787.80	1.38		1.20	
	DMCur	0.735	7384.86	1.72	1.25	1.12	2.78
	BDMcur	0.843	7921.35	2.12	1.23	1.11	3.01
Temperature							
45°C	Cur	0.701	6476.13	1.93		1.25	
	DMCur	0.800	8260.23	2.27	1.18	1.15	2.81
	BDMcur	0.902	8883.97	2.90	1.27	1.13	3.10
40°C	Cur	0.755	6516.78	1.92		1.24	
	DMCur	0.872	8094.73	2.33	1.21	1.12	2.93
	BDMcur	1.010	8892.00	3.00	1.29	1.13	3.15
50°C	Cur	0.698	5996.37	2.05		1.28	
	DMCur	0.796	8074.30	2.47	1.21	1.15	2.64
	BDMcur	0.889	8745.77	2.96	1.20	1.10	2.95

Cur: Curcumin; DMCur: Demethoxycurcumin; BDMCur: Bisdemethoxycurcumin

Table S2: Origin of plant samples and their voucher specimen

Plant sample	Specimen Registration No.	Place of collection	Date of collection	Obtained from
C. aeruginosa	PBID10021	Indonesia	2007-07-18	FPEB
C. alismatifolia	PBLA11217	Laos	2014-09-05	FPEB
C. aromatica	PBNP10297	Nepal	2013-12-31	FPEB
C. caesia	PBNP10121	Nepal	2006-10-11	FPEB
C. elata	PBCN11797	China	2008-09-09	FPEB
C. parvifolia	PBLA11321	Laos	2015-09-08	FPEB
C. heyneana	PBID10008	Indonesia	2007-07-18	FPEB
C. manga	PBID11412	Indonesia	2012-12-20	FPEB
C. phaeocaulis	PBCN11028	China	2003-09-30	FPEB
C. xanthorrhiza	PBMY10014	Malaysia	2013-09-16	FPEB
C. zedoaria	PBBG10186	Bangladesh	2013-10-16	FPEB
C. angustifolia	PBNP10043	Nepal	2006-10-11	FPEB
C. kwangsiensis	LHK035	China	2015-07-16	KPEB
C. wenyuin	LHK036	China	2015-07-16	KPEB
C. longa	PBCN10532	China	2003-09-30	FPEB
C. longa	PBID10011	Indonesia	2007-07-18	FPEB
C. longa	PBPK10014	Pakistan	2007-11-23	FPEB
C. longa	PBUS10640	USA	2008-07-29	FPEB
C. longa	PBCL10288	Chile	2009-07-10	FPEB
C. longa	PBBG10011	Bangladesh	2012-10-19	FPEB
C. longa	LHK037	Korea (Kokseong)	2015-05-06	KPEB

C. longa	LHK038	Korea (Jindo)	2015-07-01	KPEB
C. longa	LHK039	Korea (Jeju)	2015-02-24	KPEB
C. longa	PS220	India	2016-01-25	MNDRN
C. longa	PS221	Nepal (Chitawan)	2015-12-25	MNDRN
C. longa	PS222	Nepal (Sunsari)	2016-01-12	MNDRN
C. longa	PS223	Nepal (Dhangadi)	2016-01-20	MNDRN
C. longa	PS224	Nepal (Nawalparasi)	2015-12-25	MNDRN
C. longa	PS225	Nepal (Banke)	2016-01-08	MNDRN
C. longa	PS226	Nepal (Jhapa)	2015-12-01	MNDRN
C. longa	PS227	Nepal (Morang)	2015-12-03	MNDRN
C. longa	PS228	Nepal (Pyuthan)	2015-12-22	MNDRN
C. longa	PS229	Nepal (Arghakanchi)	2015-12-28	MNDRN
C. longa	PS230	Nepal (Surkhet)	2016-01-23	MNDRN
C. longa	PS231	Nepal (Dailekh)	2016-01-24	MNDRN
C. longa	PS232	Nepal (Syangja)	2015-12-15	MNDRN
C. longa	PS233	Nepal (Tanahu)	2016-01-27	MNDRN
C. longa	PS234	Nepal (Dhankuta)	2015-12-08	MNDRN
C. longa	PS235	Nepal (Ilam)	2015-12-04	MNDRN
C. longa	PS236	Nepal (Kaski)	2016-01-11	MNDRN
C. longa	PS237	Nepal (Baglung)	2016-01-12	MNDRN
C. longa	PS238	Nepal (Kalikot)	2016-01-26	MNDRN

FPEB: Foreign Plant Extract Bank
KEPB: Korea Plant Extract Bank
MNDRN: Museum of Natural Drug Resources of Nepal

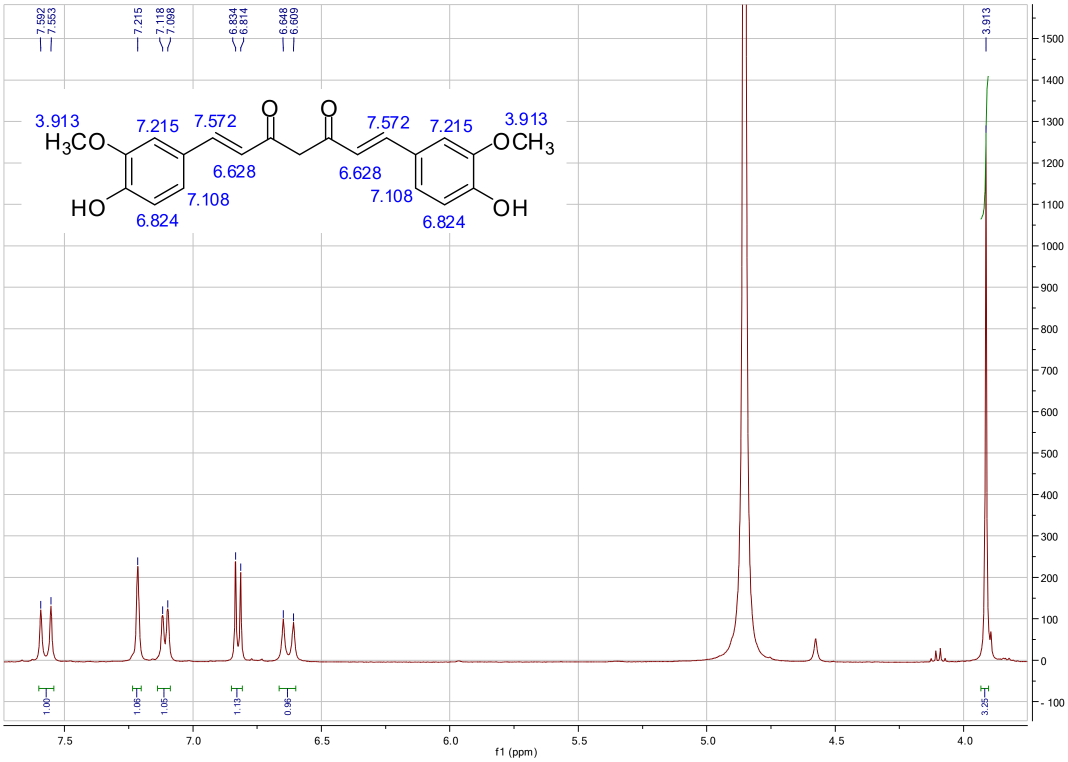

Figure S1. ¹H NMR of curcumin

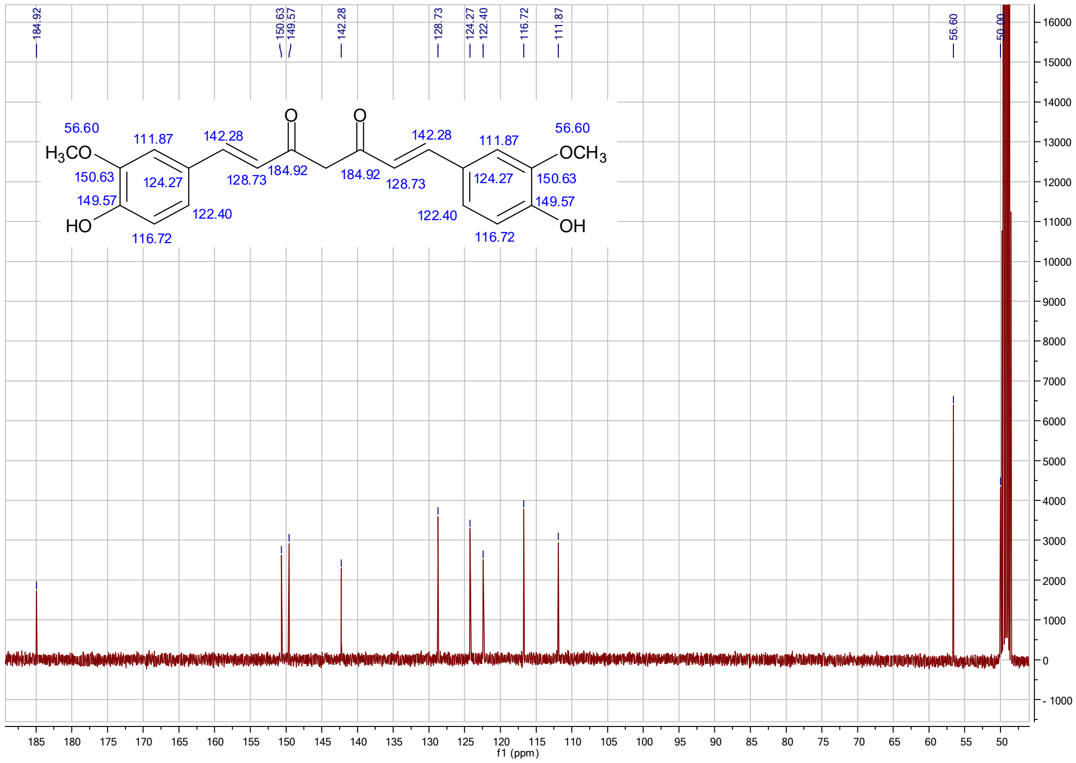

Figure S2. ^{13}C NMR of curcumin

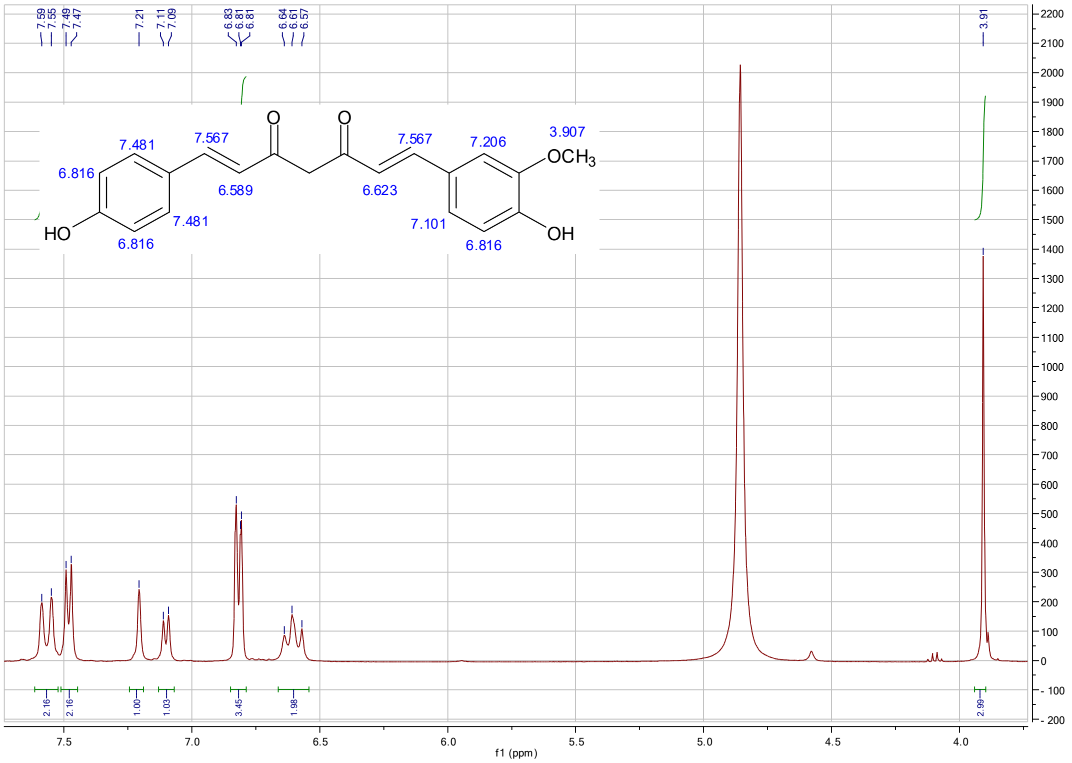

Figure S3. ^{1}H NMR of demethoxycurcumin

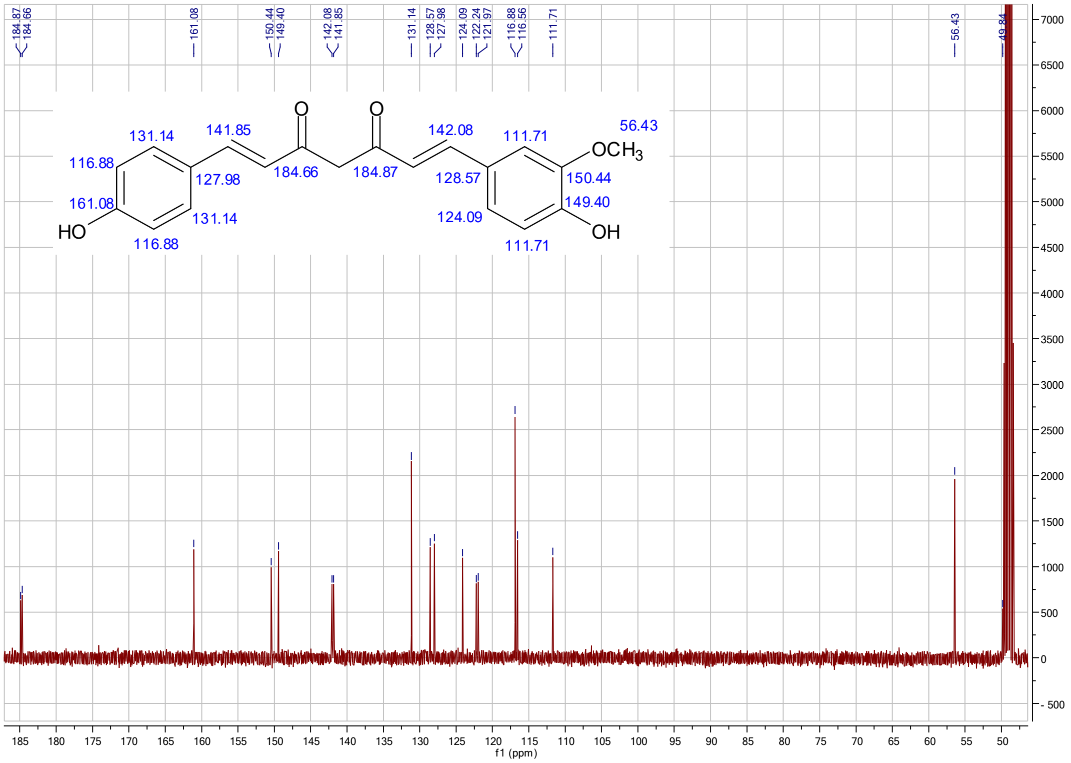

Figure S4. ^{13}C NMR of demethoxycurcumin

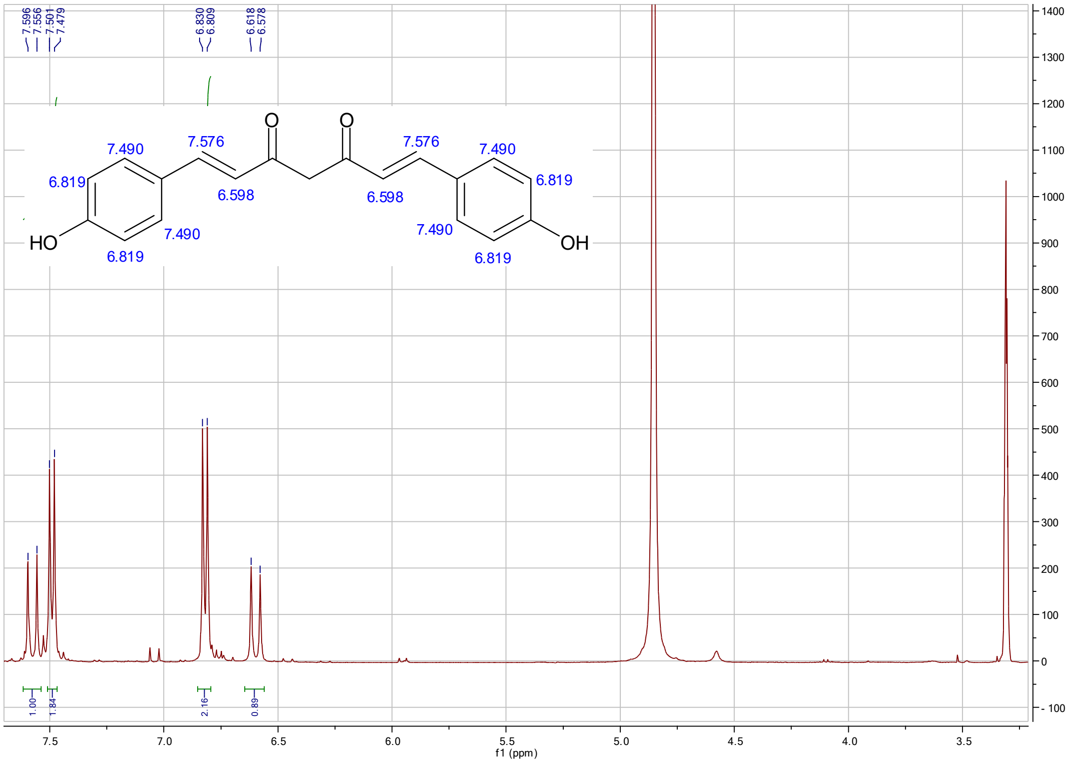

Figure S5. ^{1}H NMR of bisdemethoxycurcumin

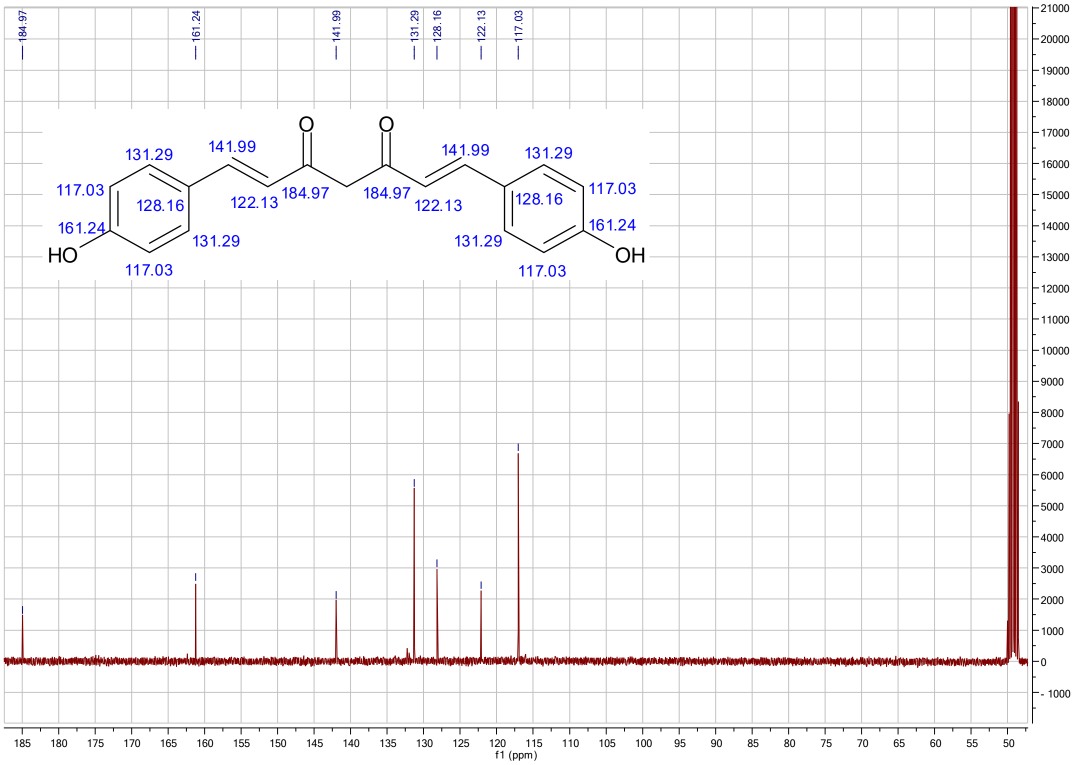

Figure S6. ^{13}C NMR of bisdemethoxycurcumin

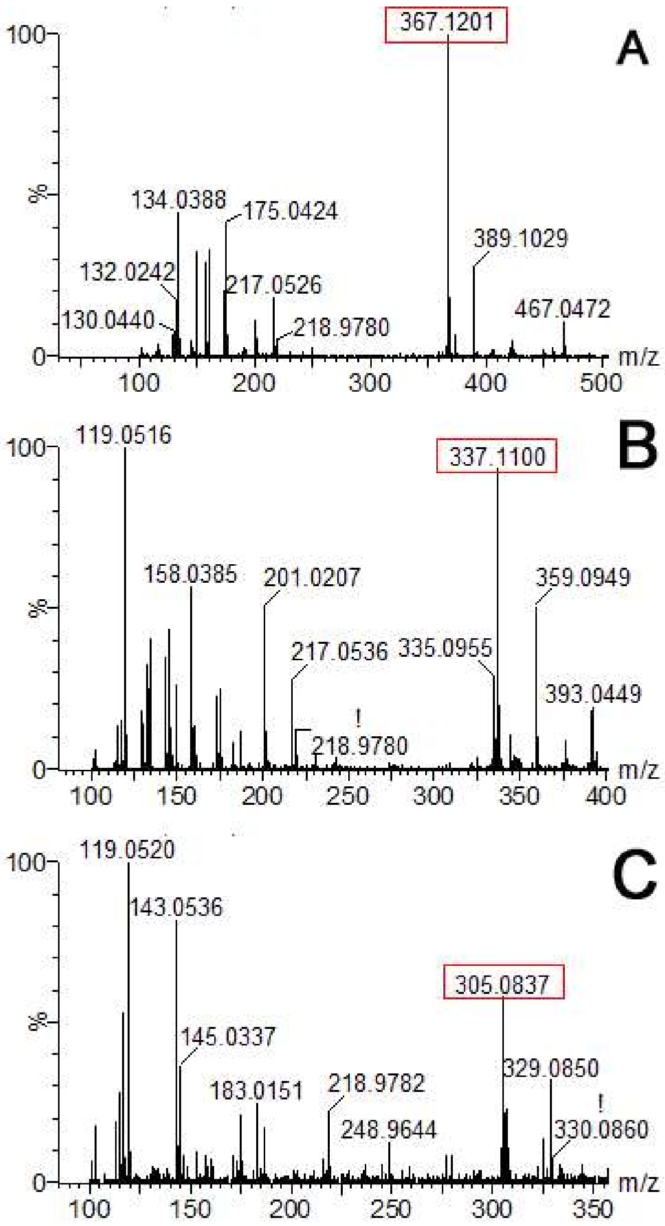

Figure S7. HR-MS [M-H] of A: Curcumin, B: Demethoxycurcumin, C: Bisdemethoxycurcumin

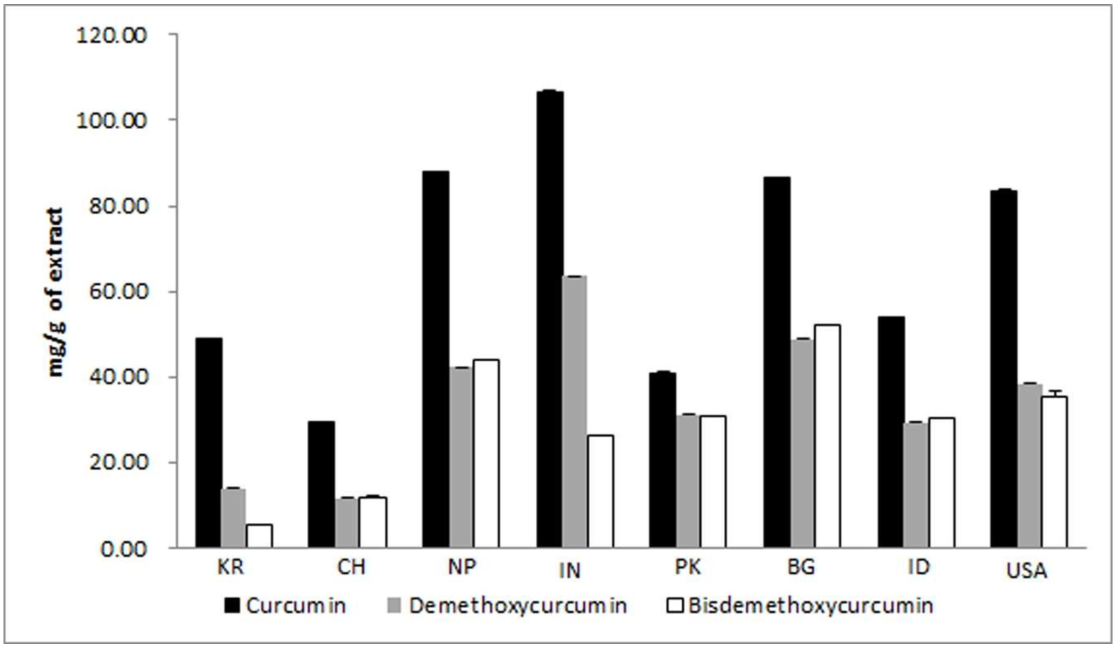

Figure S8. Curcuminoids contents of *C. longa* from different countries samples. KR: Korea; CH: China; NP: Nepal; IN: India; PK: Pakistan; BG: Bangladesh; ID: Indonesia; USA: United States of America. Values were expressed as mean±SD.
